# t-tests, non-parametric tests, and large studies—a paradox of statistical practice?

**DOI:** 10.1186/1471-2288-12-78

**Published:** 2012-06-14

**Authors:** Morten W Fagerland

**Affiliations:** 1 Unit of Biostatistics and Epidemiology, Oslo University Hospital, Oslo, N-0407, Norway

**Keywords:** T-test, Non-parametric test, Wilcoxon-Mann-Whitney test, Welch test, Sample size, Statistical practice

## Abstract

**Background:**

During the last 30 years, the median sample size of research studies published in high-impact medical journals has increased manyfold, while the use of non-parametric tests has increased at the expense of t-tests. This paper explores this paradoxical practice and illustrates its consequences.

**Methods:**

A simulation study is used to compare the rejection rates of the Wilcoxon-Mann-Whitney (WMW) test and the two-sample t-test for increasing sample size. Samples are drawn from skewed distributions with equal means and medians but with a small difference in spread. A hypothetical case study is used for illustration and motivation.

**Results:**

The WMW test produces, on average, smaller *p*-values than the t-test. This discrepancy increases with increasing sample size, skewness, and difference in spread. For heavily skewed data, the proportion of *p*<0.05 with the WMW test can be greater than 90% if the standard deviations differ by 10% and the number of observations is 1000 in each group. The high rejection rates of the WMW test should be interpreted as the power to detect that the probability that a random sample from one of the distributions is less than a random sample from the other distribution is greater than 50%.

**Conclusions:**

Non-parametric tests are most useful for small studies. Using non-parametric tests in large studies may provide answers to the wrong question, thus confusing readers. For studies with a large sample size, t-tests and their corresponding confidence intervals can and should be used even for heavily skewed data.

## Background

In an article published in the New England Journal of Medicine (NEJM) in 2005, Horton and Switzer review the use of statistical methods in three volumes of the NEJM in 2004 and 2005 [1]. They divide the methods into 25 categories—sorted according to increasing complexity—and list the frequencies in each category. Also included are the results from previous surveys of articles published in the same journal in 1978–1979 and in 1989. Table 1 presents the proportions of articles that contained t-tests and non-parametric tests. At all three time points, t-tests or non-parametric tests or both were used in more than half of the articles. In 1978–1979, four t-tests were used for every non-parametric test. In 2004–2005, t-tests and non-parametric tests were used with equal frequency.

**Table 1:** Trends in the use of t-tests and non-parametric tests in the NEJM

**Statistical procedure**	**1978–1979**	**1989**	**2004–2005**
t-tests^∗^	44%	39%	26%
Non-parametric tests^*‡*^	11%	21%	27%

^∗^one-sample, two-sample, and matched-pair [2].

^‡^Wilcoxon-Mann-Whitney, sign, and Wilcoxon signed rank sum [2].

Let us compare this trend in the use of simple statistical methods with another development. Martin Bland [3] considers the median sample size of research reports published in the Lancet and the BMJ that used individual subject data. In September 1972, the median sample sizes were 33 and 37, and in September 2007, they were 3116 and 3104. Thus, during a similar time span as in Table 1, the sample size increased almost 100 fold.

If we assume that the NEJM is similar to the Lancet and the BMJ as regards statistical methods and sample size, research authors that publish in these high-impact medical journals have increase their use of non-parametric tests at the expense of t-tests as their studies have increased in size.

This, to me, is counterintuitive.

t-tests are parametric tests, which assume that the underlying distribution of the variable of interest is normally distributed. Consider the two-sample t-test. It is fairly robust to deviations from normality [4], and—by the central limit theorem—increasingly so when the sample size increases. When the sample size of a study is 200, the t-test is robust even to heavily skewed distributions [5].

Non-parametric tests, as defined in Table 1, have, broadly speaking, two applications. First, as simple methods to analyze ordinal data, such as degree of pain classified as none, mild, moderate, or severe. Second, as alternatives to parametric tests, most often used when there is evidence of non-normality. This latter practice is advocated in many basic textbooks, such as Refs. [6-9].

In their capacity as alternatives to t-tests, non-parametric tests are thereby most useful when the sample size is small. One would, then, expect to observe an increase in the ratio of t-tests to non-parametric tests as studies grow in size. Instead, the opposite has occurred. The purpose of this paper is to illustrate the consequences of uncritical use of non-parametric tests for large studies and to discuss some possible explanations for this practice.

## Methods

Suppose that we want to compare the means or medians of a continuous variable in two independent groups. Two tests are often used for this problem: the (two-sample) t-test and the Wilcoxon-Mann-Whitney (WMW) rank sum test. The t-test is a test for the hypothesis of equal means, whereas the WMW test is less specific. If the underlying distributions of the variable in the two groups differ only in location, i.e. in means and medians, the WMW test is a test for the hypothesis of equal medians. For all other situations, the null hypothesis of the WMW test is Prob(*X*<*Y*)=0.5, where *X* and *Y * are random samples from the two distributions. Interpretation of a small *p*-value in this case is not always straightforward.

A difference in means or medians is usually accompanied by a difference in spread [10,11]. The WMW test is sensitive to distribution differences besides location [11] and may give a small *p*-value based on differences in spread even when the means and medians are equal.

A simulation study was carried out to compare the rejection rates of the t- and WMW tests for increasing sample size. Due to its superior properties [5], the t-test adjusted for unequal variances—hereafter simply referred to as the t-test, though it is often called the Welch U test—was used. The Brunner-Munzel test, a non-parametric test that adjusts for unequal variances, may be used as an alternative to the WMW test. It is not widely available in software packages, performs similarly to the WMW test [11], and is not included in the simulation study. The data were drawn at random from skewed gamma and lognormal distributions. The amount of skewness varied, in four steps, from small (coefficient of skewness = 1.0) to considerable (skewness = 4.0) and was always equal in both distributions, as were the means and medians. The only difference between the two distributions was in standard deviations, which differed, in eight steps, from 5% (ratio of 1.05) to 50% (ratio of 1.50). The nominal significance level was 5% and 10 000 replications were used.

Table 2 gives the true Prob(*X*<*Y*) for each scenario in the simulation study. Since the null hypothesis of the WMW test is Prob(*X*<*Y*)=0.5, we expect the rejection rates of the WMW test to exceed the nominal significance level whenever Prob(*X*<*Y*)>0.5. That is, the rejection rates of the WMW test represent the power to detect Prob(*X*<*Y*)≠0.5.

**Table 2:** The true Prob(X<Y) for each scenario in the simulation study

	**Gamma distributions**					**Lognormal distributions**			
	**Skewness**					**Skewness**			
**Std.ratio**	**1.0**	**2.0**	**3.0**	**4.0**		**1.0**	**2.0**	**3.0**	**4.0**
1.05	0.50	0.51	0.54	0.58		0.50	0.51	0.51	0.51
1.10	0.51	0.52	0.56	0.61		0.51	0.51	0.52	0.52
1.15	0.51	0.53	0.57	0.62		0.51	0.52	0.53	0.53
1.20	0.52	0.54	0.58	0.64		0.52	0.53	0.53	0.54
1.25	0.52	0.54	0.59	0.64		0.52	0.53	0.54	0.56
1.30	0.52	0.55	0.60	0.66		0.52	0.53	0.55	0.55
1.40	0.53	0.56	0.61	0.67		0.52	0.54	0.56	0.57
1.50	0.53	0.57	0.62	0.68		0.53	0.55	0.56	0.58

## Results

### Case study

**Figure 1:**
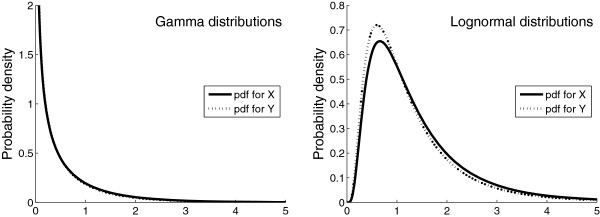
** Probability density functions (pdf) of two gamma (left panel) and two lognormal (right panel) distributions.** The two distributions in each panel are equal, except that the standard deviation of *X* is 10% greater than that of *Y *.

Consider Figure 1, which is a plot of the probability density functions of two gamma (left panel) and two lognormal (right panel) distributions. The coefficient of skewness is 3.0 for all distributions, and the means and the medians of the two distributions in each panel are equal. The standard deviation of the distributions corresponding to the solid lines (*X*) are 10% greater than that of the distributions corresponding to the dotted lines (*Y *). That difference is almost imperceptible for the two gamma distributions.

**Figure 2:**
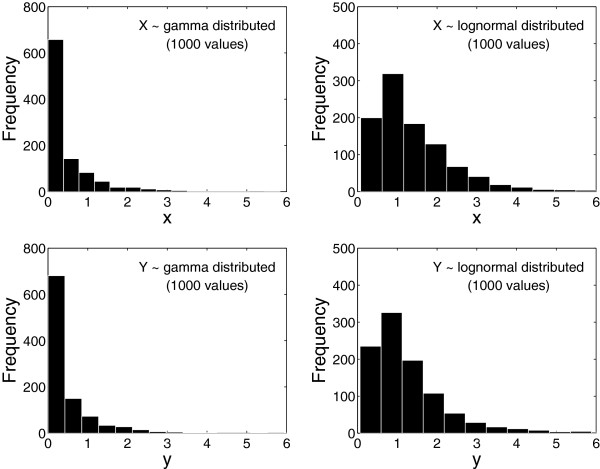
** Histograms of random samples of size 1000 drawn from the four distributions in Figure **1**.**

Suppose that we draw, at random, 1000 values from each of these four distributions. The results might look like that of Figure 2. Since we, in an actual study, obviously do not know the exact distributions from which the observed data originate, it is histograms such as these that give us a clue about the underlying distributions of the data. The data in Figure 2 are markedly skewed to the right, and we may be tempted to use the WMW test instead of the t-test to compare the locations of *X* and *Y *.

If we repeatedly draw samples of size 1000 from the distributions in Figure 1, we can apply the t- and WMW tests to the samples each time and record the results. After 10 000 replications, the 5% rejection rates (the proportion of times *p*<0.05) were 5.1% (gamma distributions) and 4.9% (lognormal distributions) for the t-test. The expected rejection rate for an unbiased test of means or medians is 5.0%, that is, a one in 20 chance of a significant result when the means (and medians) are known to be equal. The t-test thus performs quite well. The rejection rates for the WMW test are 99% (gamma distributions) and 28% (lognormal distributions). The WMW test indicates a significant difference between the groups more often than the expected 5%. The explanation is that the two distributions are slightly different: their means and medians are equal but their standard deviations differ by 10%. The WMW test is sensitive to this difference and produces a small *p*-value. But, if we are interested in comparing the means or the medians—as is customary—the WMW test most likely gives us an answer to the wrong question. The correct question for the WMW test can be formulated as: Is a random sample from one of the distributions likely to be less than a random sample from the other distribution? The skewness and standard deviation ratio of the two distributions in Figure 1 are 3.0 and 1.10, respectively. We thereby obtain the actual probability of Prob(*X*<*Y*) from Table 2, which is 56% for the gamma distributions and 52% for the lognormal distributions. The high rejection rates of the WMW test (99% and 28%) represent the power of the WMW test to detect that those probabilities are unequal to 50%.

**Figure 3:**
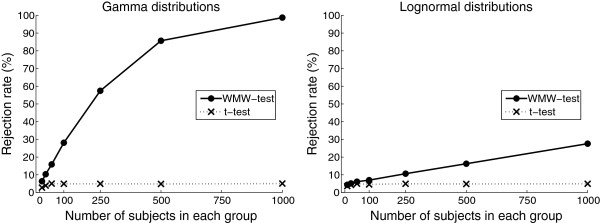
** Rejection rates (*p*<***0.05***) of the WMW and t-tests based on samples from the distributions in Figure**1**.**

If we repeat the above exercise for a range of sample size values, we can plot the rejection rate against the number of subjects in each group (Figure 3). The rejection rates of the WMW test increase as the sample size increases, whereas the rejection rates of the t-test are stable at about 5%.

### Overall results from the simulation study

The patterns of rejections rates in Figure 3 persist for all combinations of skewness and standard deviation ratios considered in this study. The rejection rates of the t-test are always close to 5%, whereas the rejection rates of the WMW test increases with increasing sample size. As expected, the rejection rates of the WMW test increases when the difference in standard deviations increases, because it is this difference that the WMW test picks up. Interestingly, the rejection rates of the WMW test also increases when the amount of skewness increases. The problem is thus greater for situations in which one would more readily abandon the t-test (considerably skewed data) than for situations where the amount of skewness may be considered manageable (slightly skewed data). An example of the increasing rejection rates of the WMW test for increasing standard deviation ratios and increasing amount of skewness can be seen in Table 3.

**Table 3:** Rejection rates (%) of the t- and WMW tests for data drawn from gamma distributions using 1000 subjects in each group

	**WMW test**					**t-test**			
	**Skewness**					**Skewness**			
**Std.ratio**	**1.0**	**2.0**	**3.0**	**4.0**		**1.0**	**2.0**	**3.0**	**4.0**
1.05	5.8	14.4	78.6	100		4.8	4.4	4.9	4.8
1.10	8.6	39.1	98.8	100		4.8	5.0	5.1	4.9
1.15	13.6	65.7	100	100		5.5	4.8	5.1	5.2
1.20	19.6	83.7	100	100		5.0	4.7	5.2	5.2
1.25	26.1	93.8	100	100		5.7	4.7	5.0	5.1
1.30	33.9	97.7	100	100		4.9	5.0	4.4	5.1
1.40	48.9	99.8	100	100		4.9	5.0	5.0	4.8
1.50	61.7	100	100	100		4.7	5.0	4.9	5.3

Detailed results for each of the 448 situations considered in the simulation study are given in Additional file 1. Tables 4 and 5 present summaries of the results. In Table 4, the average per cent rejection rates of the t- and WMW tests are given stratified by study size. Each value in the table is the mean of the rejection rates for each of the 32 combinations of amount of skewness and standard deviation ratios.

**Table 4:** Mean rejection rates (%) of the t- and WMW tests, averaged over 32 combinations of amount of skewness and standard deviation ratios

	**Number of subjects in each group**						
	**10**	**25**	**50**	**100**	**250**	**500**	**1000**
Gamma distributions							
t-test	4.01	4.71	4.91	4.98	4.95	4.99	4.97
WMW test	9.47	18.0	28.9	41.6	56.9	66.3	74.7
Lognormal distributions							
t-test	4.20	4.69	4.83	4.89	4.95	4.92	4.98
WMW test	5.21	6.99	9.63	14.4	26.5	39.7	54.3

**Table 5:** Estimated probabilities (%) that the *p*-value of the WMW test is smaller than that of the t-test, averaged over 32 combinations of amount of skewness and standard deviation ratios

	**Number of subjects in each group**						
	**10**	**25**	**50**	**100**	**250**	**500**	**1000**
Gamma distributions	54.1	63.3	69.8	75.8	82.6	86.8	90.3
Lognormal distributions	45.6	52.1	56.9	62.3	70.1	76.3	82.5

Table 5 presents the estimated probability that the *p*-value of the WMW test is smaller than that of the t-test. For large studies with data distributed as in this simulation study, the WMW test almost always produces smaller *p*-values than the t-test.

## Discussion

The concurrent increases—since the Seventies—in sample size and use of non-parametric tests over t-tests have a paradoxical quality. The usefulness of non-parametric tests as alternatives to t-tests for non-normally distributed data is most pronounced for small studies. When the sample size increases, so does the robustness of the t-tests to deviations from normality. The non-parametric WMW test, on the other hand, increases its sensitivity to distribution differences other than between means and medians, and it may detect (i.e. produce a small *p*-value) slight differences in spread. When the difference in spread increases, the probability that a random sample from one of the distributions is less than a random sample from the other distribution also increases. With a large sample size, the WMW test has great power to detect that that probability is not 50%. If the purpose of the study is to detect any distributional difference, using a non-parametric test is probably useful. Most studies, however, are carried out to investigate differences in means or medians, and as such, the ratio of non-parametric tests to t-tests ought to decrease when studies grow in size.

Why then has the use of non-parametric tests increased? We may propose several explanations. Perhaps, non-parametric tests were underused earlier, and that the present ratio of t-tests to non-parametric tests represents the “correct” one. If so, only the smallest of contemporary studies ought to use non-parametric tests. However, in the NEJM in 2004–2005, 27% of the studies used non-parametric tests [1], and the 25th percentile of the sample size in September 2007 in the Lancet and the BMJ were 1236 and 236 [3]. The smallest quartile of studies actually contains many quite large studies. Thus, the use of non-parametric tests is not confined to appropriately small studies. Another explanation might be that most studies do not use non-parametric tests as an alternative to t-tests but rather to analyze ordinal variables, which is a highly reasonable practice. We do not have any systematic evidence to support or reject that hypothesis, although a cursory review of articles published in the NEJM, Lancet, JAMA, and BMJ from September through November 2011 revealed several large studies that used non-parametric tests as alternatives to t-tests; for example, *n*=1721 [12], *n*=429 [13], *n*=107018 [14], *n*=44350 [15], *n*=1789 [16], and *n*=12745 [17]. The use of non-parametric tests as alternatives to t-tests may be more common in high-impact journals [18]. The NEJM, for instance, in their instructions for authors, recommend that “nonparametric methods should be used to compare groups when the distribution of the dependent variable is not normal” ( , accessed March 19, 2012). That recommendation does not take into account the sample size and may force authors of large studies to use non-parametric methods needlessly. Four more explanations can be hypothesized. First, medical research authors may use a test for normality to decide whether to use a t-test or a non-parametric test. We strongly advise against that practice. In large studies, tests for normality are very sensitive to deviations from normality and thereby unsuitable as tools to choose the most appropriate test. Second, regardless of the size of their studies, authors may rely on recommendations and advice intended solely for the analysis of smaller studies. There might be a lack of critical thinking about recommendations and a poor understanding of the practical implications of the central limit theorem. Third, authors may simply prefer small *p*-values, and might go shopping for the statistical method that gives them the smallest *p*. In the simulation study in this paper, the WMW test produced smaller *p*-values that the t-test more than 70% of the times when the number of subjects in each group was 250. For 1000 subjects in each group, that proportion increased to more than 80%. Last, we have publication bias. A study with a significant *p*-value from the WMW test may be more readily accepted for publication than a study with a non-significant *p*-value from the t-test.

Is the WMW test a bad test? No, but it is not always an appropriate alternative to the t-test. The WMW test is most useful for the analysis of ordinal data and may also be used in smaller studies, under certain conditions, to compare means or medians [5,11]. Furthermore, if the results from the WMW test are interpreted strictly according to the test’s null hypothesis, Prob(*X*<*Y*)=0.5, the WMW test is an efficient and useful test. For large studies, however, where the purpose is to compare the means of continuous variables, the choice of test is easy: the t-test is robust even to severely skewed data and should be used almost exclusively.

One further benefit of using the t-test is that it facilitates interval estimation. The t-test and its corresponding confidence interval are based on the same standard error estimate; when the t-test is robust, so is the confidence interval. Combined with linear regression analysis, the t-test and its confidence interval form a simple and unified approach for analyzing and presenting continuous outcome data, which, for large studies, is sufficient for most practical purposes.

This study has only considered smooth, skewed distributions. Medical variables do not always have a smooth distribution and may include outliers. The problem with outliers is not that the t-test fails as a test of equality of means in their presence, but that the mean itself may be a poor representation of the typical value of the distribution. One solution is to use another measure of location, for instance, the trimmed mean, which may be compared in two groups with the Yuen-Welch test [5]. The problem that the mean does not reflect the central tendency of a distribution is most pronounced in small studies, where the impact of outliers is usually greater than in large studies.

## Conclusions

The use of non-parametric tests in high-impact medical journals has increased at the expense of t-tests, while the sample size of research studies has increased manyfold. Recent examples of large studies that use non-parametric tests as alternatives to t-tests are abundant.

Non-parametric tests are most useful for small studies. Research authors that use non-parametric tests in large studies may provide answers to the wrong question, thus confusing readers. For large studies, t-tests and their corresponding confidence intervals can and should be used even for heavily skewed data.

## Competing interests

The author declares no competing interests.

## Pre-publication history

The pre-publication history for this paper can be accessed here:

http://www.biomedcentral.com/1471-2288/12/78/prepub

## Supplementary Material

Additional file 1 Supplementary materials. Detailed results from the simulation study. Click here for file
