# Supplementary material for: t-tests, non-parametric tests, and large studies—a paradox of statistical practice?
Source: BMC Med Res Methodol. 2012 Jun 14;12:78. doi: 10.1186/1471-2288-12-78 (PMC3445820; doi:10.1186/1471-2288-12-78)
Supplement: Additional file 1 — Supplementary materials. Detailed results from the simulation study. [file 1471-2288-12-78-S1.pdf]

Supplementary materials

t-tests, non-parametric tests, and large studies—a paradox of statistical practice?

Morten W. Fagerland

Unit of Biostatistics and Epidemiology, Oslo University Hospital, Norway

Explanation for the color codings in the tables

Tables 1.1, 2.1, 3.1, ...

- Green cells: the rejection rates (rr) are within 10% of the nominal level ($4.5 \leq rr \leq 5.5$)
- Yellow cells: the rejection rates are within 20% of the nominal level ($4.0 \leq rr \leq 6.0$)
- Red cells: the rejection rates differ by more than 20% from the nominal level ($rr < 4.0$ or $rr > 6.0$)

Tables 1.2, 2.2, 3.2, ...

- Green cells: the probabilities (p) are within 10% of 50% ($45 \leq p \leq 55$)
- Yellow cells: the probabilities are within 20% of 50% ($40 \leq p \leq 60$)
- Red cells: the probabilities differ by more than 20% from 50% ($p < 40$ or $p > 60$)

Content	Page
Tables of rejection rates and $\Pr(p_W < p_t)^*$ for 10 subjects in each group	2
Tables of rejection rates and $\Pr(p_W < p_t)^*$ for 25 subjects in each group	3
Tables of rejection rates and $\Pr(p_W < p_t)^*$ for 50 subjects in each group	4
Tables of rejection rates and $\Pr(p_W < p_t)^*$ for 100 subjects in each group	5
Tables of rejection rates and $\Pr(p_W < p_t)^*$ for 250 subjects in each group	6
Tables of rejection rates and $\Pr(p_W < p_t)^*$ for 500 subjects in each group	7
Tables of rejection rates and $\Pr(p_W < p_t)^*$ for 1000 subjects in each group	8
Plot of rejection rates against sample size for stdratio† = 1.05 and skewness = $1, 2, 3, 4$	9, 10
Plot of rejection rates against sample size for stdratio† = 1.10 and skewness = $1, 2, 3, 4$	11, 12
Plot of rejection rates against sample size for stdratio† = 1.15 and skewness = $1, 2, 3, 4$	13, 14
Plot of rejection rates against sample size for stdratio† = 1.20 and skewness = $1, 2, 3, 4$	15, 16
Plot of rejection rates against sample size for stdratio† = 1.25 and skewness = $1, 2, 3, 4$	17, 18
Plot of rejection rates against sample size for stdratio† = 1.30 and skewness = $1, 2, 3, 4$	19, 20
Plot of rejection rates against sample size for stdratio† = 1.40 and skewness = $1, 2, 3, 4$	21, 22
Plot of rejection rates against sample size for stdratio† = 1.50 and skewness = $1, 2, 3, 4$	23, 24

*Estimated probability that the p-value of the WMW test is smaller than that of the t-test

†Standard deviation ratio

10 subjects in each group

gamma distributions				WMW test	lognormal distributions			
				std.ratio				
5.1	8.2	14.9	25.9	1.50	5.4	6.0	7.2	8.5
4.8	6.6	13.1	23.7	1.40	4.7	6.1	6.6	7.8
5.1	5.9	12.1	21.5	1.30	4.4	5.3	5.5	6.1
4.7	5.6	10.2	19.5	1.25	4.5	4.9	5.2	5.7
4.5	5.5	8.2	17.1	1.20	4.6	4.6	4.7	5.7
4.4	5.0	7.4	14.7	1.15	4.3	4.7	4.7	4.7
4.5	4.5	6.3	12.1	1.10	4.2	4.2	4.3	4.9
4.3	4.3	5.1	8.5	1.05	4.1	4.3	3.9	4.6
1.0	2.0	3.0	4.0	skewness	1.0	2.0	3.0	4.0

gamma distributions				t-test	lognormal distributions			
				std.ratio				
5.1	5.0	5.3	6.0	1.50	5.5	4.9	4.9	4.5
4.6	4.5	4.9	4.5	1.40	4.9	4.8	4.8	4.4
5.0	4.0	3.9	4.1	1.30	4.4	4.6	3.8	3.8
4.8	4.1	3.6	3.4	1.25	5.1	4.2	3.8	3.6
4.3	4.2	3.3	2.9	1.20	4.7	3.9	3.7	3.8
4.7	4.1	2.9	2.5	1.15	4.3	4.2	3.9	3.2
4.7	3.9	2.8	2.2	1.10	4.5	3.9	3.6	3.4
4.5	3.7	2.8	2.1	1.05	4.4	3.8	3.5	3.4
1.0	2.0	3.0	4.0	skewness	1.0	2.0	3.0	4.0

Table 1.1: Rejection rates (%).

gamma distributions					lognormal distributions			
				std.ratio				
46.4	53.6	65.6	74.8	1.50	45.3	47.9	50.1	54.1
45.0	51.7	63.1	73.3	1.40	44.0	46.6	49.6	51.5
45.1	50.0	60.5	71.5	1.30	43.4	45.8	46.1	49.6
43.7	48.6	58.8	69.7	1.25	44.1	45.1	46.5	47.7
43.5	47.3	56.3	67.9	1.20	43.1	43.4	45.3	45.6
42.2	46.1	54.1	64.7	1.15	42.6	43.2	44.5	45.6
42.9	45.2	51.0	60.9	1.10	42.6	42.6	43.8	43.6
42.6	43.7	47.3	55.4	1.05	43.4	43.3	42.9	43.0
1.0	2.0	3.0	4.0	skewness	1.0	2.0	3.0	4.0

Table 1.2: Estimated probabilities (%) that the p-value of the WMW test is smaller than that of the t-test.

25 subjects in each group

gamma distributions				WMW test	lognormal distributions			
				std.ratio				
6.7	13.5	32.7	58.4	1.50	6.0	9.0	11.5	16.2
5.8	11.2	28.5	54.3	1.40	5.9	7.9	10.0	13.3
5.3	8.9	24.2	48.1	1.30	5.5	6.3	8.1	9.6
5.5	8.2	19.9	43.1	1.25	5.0	6.3	7.7	8.2
5.4	7.2	16.8	38.3	1.20	5.1	5.3	6.1	7.5
4.8	6.3	12.8	32.0	1.15	5.3	5.3	6.5	6.3
5.1	5.6	10.3	24.7	1.10	4.5	5.1	5.1	5.7
4.6	5.2	6.4	15.5	1.05	4.6	4.8	5.1	4.8
1.0	2.0	3.0	4.0	skewness	1.0	2.0	3.0	4.0

gamma distributions				t-test	lognormal distributions			
				std.ratio				
5.3	5.3	5.7	5.7	1.50	5.1	5.1	4.9	5.1
4.6	5.0	5.2	4.8	1.40	4.9	5.2	4.8	4.9
4.7	4.6	5.0	4.9	1.30	5.0	4.6	4.4	4.7
5.1	4.4	4.7	4.7	1.25	4.7	5.3	4.5	4.5
5.1	5.0	4.8	3.7	1.20	4.9	4.6	4.4	4.3
4.7	4.7	4.1	3.9	1.15	4.9	4.8	4.3	4.2
5.2	4.6	3.9	3.8	1.10	4.6	4.6	4.3	4.3
4.7	4.8	4.0	4.0	1.05	5.2	4.7	4.4	3.8
1.0	2.0	3.0	4.0	skewness	1.0	2.0	3.0	4.0

Table 2.1: Rejection rates (%).

gamma distributions					lognormal distributions			
				std.ratio				
51.7	64.4	79.9	88.5	1.50	52.4	57.9	61.8	66.4
51.8	62.4	76.2	86.8	1.40	50.6	54.7	58.4	62.4
50.1	58.9	73.5	84.9	1.30	49.1	52.0	55.3	59.2
49.5	56.6	70.8	83.1	1.25	48.2	51.8	53.4	55.9
48.4	55.3	67.8	81.0	1.20	47.7	49.8	50.5	53.2
47.9	51.6	63.5	77.6	1.15	48.2	48.6	50.5	51.2
47.4	50.3	58.7	72.8	1.10	47.3	47.9	48.3	48.3
47.6	48.4	53.2	63.9	1.05	46.3	47.0	46.6	47.5
1.0	2.0	3.0	4.0	skewness	1.0	2.0	3.0	4.0

Table 2.2: Estimated probabilities (%) that the p-value of the WMW test is smaller than that of the t-test.

50 subjects in each group

gamma distributions				WMW test	lognormal distributions			
				std.ratio				
8.6	24.1	58.3	86.5	1.50	7.5	13.8	20.6	28.2
7.6	18.6	50.7	82.4	1.40	6.7	10.7	16.4	21.9
6.1	14.2	40.8	76.0	1.30	6.1	8.6	12.3	16.3
6.5	12.5	35.5	71.2	1.25	5.5	7.7	10.4	13.4
5.6	10.5	30.1	64.9	1.20	5.5	6.9	8.5	10.5
5.5	8.0	23.2	56.5	1.15	5.4	6.1	7.2	8.6
5.1	6.7	15.9	45.6	1.10	4.9	5.4	6.1	6.5
5.3	5.6	9.9	28.1	1.05	4.9	4.6	5.5	5.5
1.0	2.0	3.0	4.0	skewness	1.0	2.0	3.0	4.0

gamma distributions				t-test	lognormal distributions			
				std.ratio				
5.3	5.2	5.4	5.1	1.50	4.7	4.8	5.1	5.2
5.0	5.0	5.5	5.2	1.40	4.9	5.0	5.0	5.4
4.7	4.7	4.8	5.0	1.30	4.9	5.1	4.7	4.9
5.3	5.0	4.9	4.7	1.25	4.7	4.7	4.8	5.1
5.1	4.9	4.6	4.2	1.20	5.0	5.0	4.7	4.6
5.0	5.0	4.7	4.6	1.15	5.1	4.7	4.7	4.8
4.9	5.0	5.0	4.4	1.10	4.5	4.8	5.0	4.3
5.1	4.8	4.7	4.4	1.05	5.0	4.5	4.7	4.4
1.0	2.0	3.0	4.0	skewness	1.0	2.0	3.0	4.0

Table 3.1: Rejection rates (%).

gamma distributions					lognormal distributions			
				std.ratio				
57.8	73.6	87.7	95.8	1.50	56.1	64.5	71.5	76.6
56.2	70.5	85.5	94.6	1.40	54.7	60.7	67.5	72.0
54.1	66.1	81.8	93.3	1.30	53.1	57.8	61.6	66.6
52.5	63.5	79.8	91.9	1.25	51.6	56.4	60.1	63.2
51.9	61.0	77.8	90.4	1.20	50.5	54.3	56.8	59.9
50.2	57.9	72.2	87.0	1.15	50.2	52.0	53.3	54.6
48.5	52.5	65.7	83.4	1.10	49.2	49.3	51.5	52.0
49.5	49.9	57.5	74.3	1.05	48.0	48.4	48.6	49.5
1.0	2.0	3.0	4.0	skewness	1.0	2.0	3.0	4.0

Table 3.2: Estimated probabilities (%) that the p-value of the WMW test is smaller than that of the t-test.

100 subjects in each group

gamma distributions				WMW test	lognormal distributions			
				std.ratio				
11.7	40.4	84.5	99.0	1.50	9.6	22.9	36.8	48.6
10.2	32.7	79.2	98.2	1.40	8.5	17.2	27.6	37.7
7.8	24.4	68.7	96.3	1.30	7.5	12.9	20.4	27.2
7.4	20.6	61.2	94.3	1.25	6.3	11.0	16.0	22.3
6.3	15.3	51.8	90.9	1.20	6.1	8.8	12.3	17.7
5.5	11.6	40.8	84.6	1.15	6.0	7.3	9.4	12.3
5.1	8.4	28.1	73.1	1.10	5.6	5.6	7.0	8.2
5.0	6.0	14.1	48.7	1.05	5.1	5.0	5.8	5.9
1.0	2.0	3.0	4.0	skewness	1.0	2.0	3.0	4.0

gamma distributions				t-test	lognormal distributions			
				std.ratio				
5.1	5.1	5.2	5.0	1.50	4.8	5.1	5.1	5.2
5.0	4.9	5.0	5.0	1.40	4.6	4.6	4.8	4.9
5.1	5.2	5.0	4.9	1.30	5.0	5.1	5.2	4.6
4.9	5.3	4.8	4.8	1.25	4.7	4.9	4.7	4.8
4.6	5.2	5.2	4.8	1.20	4.8	5.1	4.9	4.5
5.0	5.4	5.0	5.0	1.15	5.1	5.0	4.8	5.0
4.7	5.3	4.9	4.7	1.10	5.3	4.7	4.6	4.8
4.9	5.0	4.7	4.7	1.05	5.2	4.8	5.0	4.8
1.0	2.0	3.0	4.0	skewness	1.0	2.0	3.0	4.0

Table 4.1: Rejection rates (%).

gamma distributions					lognormal distributions			
				std.ratio				
64.2	82.0	94.8	99.3	1.50	61.4	72.4	80.3	85.2
61.2	78.5	93.4	99.0	1.40	59.6	69.5	75.8	81.0
58.3	73.4	90.8	98.1	1.30	56.6	64.0	70.4	76.2
56.1	70.6	88.7	97.4	1.25	54.1	62.0	67.3	72.0
54.2	67.3	85.2	96.6	1.20	53.9	58.5	63.9	67.6
52.5	62.4	81.8	94.7	1.15	51.7	55.8	58.7	61.5
51.0	58.0	75.1	92.0	1.10	50.6	52.0	53.7	55.4
50.6	51.8	63.9	84.3	1.05	50.4	50.1	50.3	50.8
1.0	2.0	3.0	4.0	skewness	1.0	2.0	3.0	4.0

Table 4.2: Estimated probabilities (%) that the p-value of the WMW test is smaller than that of the t-test.

250 subjects in each group

gamma distributions				WMW test	lognormal distributions			
				std.ratio				
20.4	76.9	99.7	100.0	1.50	17.6	46.5	71.0	86.0
15.9	67.2	99.2	100.0	1.40	14.1	36.0	58.4	75.0
11.8	50.6	96.9	100.0	1.30	11.3	24.7	42.1	56.9
10.0	41.9	94.2	100.0	1.25	8.6	19.5	33.6	45.8
8.4	31.6	88.9	99.9	1.20	7.6	14.9	24.3	34.4
6.9	21.8	76.8	99.5	1.15	6.7	11.0	16.7	22.9
6.0	12.7	57.5	98.0	1.10	6.0	7.8	10.6	13.5
5.5	7.3	28.5	85.9	1.05	5.4	5.9	6.5	7.2
1.0	2.0	3.0	4.0	skewness	1.0	2.0	3.0	4.0

gamma distributions				t-test	lognormal distributions			
				std.ratio				
4.8	5.0	5.4	5.2	1.50	5.1	5.2	5.1	4.9
4.9	5.0	4.8	4.9	1.40	4.8	5.0	5.1	5.0
5.0	5.0	5.3	4.5	1.30	4.8	4.5	4.5	4.7
4.9	5.1	5.2	4.9	1.25	4.6	4.9	5.2	4.8
4.8	4.8	4.8	4.9	1.20	5.0	5.0	5.1	4.8
4.8	5.1	5.0	5.0	1.15	4.7	5.3	5.5	4.9
5.0	4.8	4.9	4.8	1.10	5.0	4.8	4.9	5.0
5.3	5.1	4.7	4.7	1.05	5.0	5.0	5.0	4.7
1.0	2.0	3.0	4.0	skewness	1.0	2.0	3.0	4.0

Table 5.1: Rejection rates (%).

gamma distributions					lognormal distributions			
				std.ratio				
71.8	92.0	99.5	100.0	1.50	69.6	83.0	90.6	94.9
68.6	89.5	99.2	100.0	1.40	66.5	79.6	87.2	92.2
64.8	84.5	98.1	100.0	1.30	63.1	74.2	81.7	87.8
62.3	81.3	97.1	100.0	1.25	59.9	71.2	78.9	83.4
59.4	77.1	95.7	99.8	1.20	58.4	66.1	74.2	79.1
55.9	72.3	92.5	99.5	1.15	56.0	61.9	67.4	72.4
53.6	65.2	87.5	98.6	1.10	52.6	56.7	60.8	63.6
50.3	56.2	75.6	95.3	1.05	51.0	51.9	52.3	54.5
1.0	2.0	3.0	4.0	skewness	1.0	2.0	3.0	4.0

Table 5.2: Estimated probabilities (%) that the p-value of the WMW test is smaller than that of the t-test.

500 subjects in each group

gamma distributions				WMW test	lognormal distributions			
				std.ratio				
35.6	96.7	100.0	100.0	1.50	29.6	75.1	94.3	98.8
27.3	91.6	100.0	100.0	1.40	23.1	61.3	86.1	96.1
19.4	80.4	100.0	100.0	1.30	15.7	42.9	70.4	85.5
15.2	69.2	99.8	100.0	1.25	12.8	33.0	57.3	74.5
12.3	55.5	99.3	100.0	1.20	10.4	24.3	42.7	59.0
8.5	38.6	96.8	100.0	1.15	8.2	16.7	28.7	40.5
6.7	22.6	85.7	100.0	1.10	6.1	10.4	16.3	22.2
5.3	9.2	48.6	98.9	1.05	5.2	6.5	8.2	9.5
1.0	2.0	3.0	4.0	skewness	1.0	2.0	3.0	4.0

gamma distributions				t-test	lognormal distributions			
				std.ratio				
4.7	5.2	5.1	4.5	1.50	4.6	4.9	5.1	4.9
4.9	5.0	4.9	5.2	1.40	5.2	5.0	5.0	5.1
5.1	4.8	5.0	4.8	1.30	4.6	5.0	4.7	5.0
5.2	5.0	5.0	5.5	1.25	4.9	5.0	4.4	5.2
4.6	5.0	4.9	5.1	1.20	5.1	5.1	4.6	5.0
4.8	5.4	5.7	4.8	1.15	5.1	4.5	5.3	4.7
4.7	5.2	4.8	4.7	1.10	4.5	5.2	4.8	4.8
4.8	5.1	5.1	5.0	1.05	4.9	5.1	5.2	4.9
1.0	2.0	3.0	4.0	skewness	1.0	2.0	3.0	4.0

Table 6.1: Rejection rates (%).

gamma distributions					lognormal distributions			
				std.ratio				
79.3	97.7	100.0	100.0	1.50	76.9	91.7	97.0	98.9
74.9	95.7	100.0	100.0	1.40	72.7	87.7	94.8	97.8
71.2	92.9	99.9	100.0	1.30	69.3	81.7	90.4	94.8
67.8	90.4	99.6	100.0	1.25	65.2	77.7	87.3	91.7
64.7	86.0	99.3	100.0	1.20	62.7	74.3	82.6	87.8
61.2	80.2	97.7	100.0	1.15	58.9	68.6	76.6	81.8
56.2	72.1	95.0	99.9	1.10	54.2	61.5	67.3	71.6
51.6	59.7	85.3	99.2	1.05	52.1	53.7	54.5	58.1
1.0	2.0	3.0	4.0	skewness	1.0	2.0	3.0	4.0

Table 6.2: Estimated probabilities (%) that the p-value of the WMW test is smaller than that of the t-test.

1000 subjects in each group

gamma distributions				WMW test	lognormal distributions			
				std.ratio				
61.7	100.0	100.0	100.0	1.50	52.9	95.6	99.9	100.0
48.9	99.8	100.0	100.0	1.40	39.9	88.6	99.1	99.9
33.9	97.7	100.0	100.0	1.30	27.4	71.8	93.7	99.1
26.1	93.8	100.0	100.0	1.25	21.4	59.2	85.3	96.2
19.6	83.7	100.0	100.0	1.20	16.4	44.5	71.0	87.3
13.6	65.7	100.0	100.0	1.15	11.9	28.4	49.8	67.6
8.6	39.1	98.8	100.0	1.10	8.0	15.6	27.6	39.5
5.8	14.4	78.6	100.0	1.05	6.1	7.8	10.3	14.5
1.0	2.0	3.0	4.0	skewness	1.0	2.0	3.0	4.0

gamma distributions				t-test	lognormal distributions			
				std.ratio				
4.7	5.0	4.9	5.3	1.50	4.7	4.9	5.0	5.0
4.9	5.0	5.0	4.8	1.40	4.8	5.1	5.1	4.8
4.9	5.0	4.4	5.1	1.30	4.9	4.8	5.2	5.2
5.7	4.7	5.0	5.1	1.25	5.0	5.2	4.7	4.9
5.0	4.7	5.2	5.2	1.20	5.1	4.8	5.1	4.7
5.5	4.8	5.1	5.2	1.15	4.8	5.1	5.2	5.1
4.8	5.0	5.1	4.9	1.10	5.2	5.0	4.9	5.2
4.8	4.4	4.9	4.8	1.05	5.1	4.8	5.0	4.9
1.0	2.0	3.0	4.0	skewness	1.0	2.0	3.0	4.0

Table 7.1: Rejection rates (%).

gamma distributions					lognormal distributions			
				std.ratio				
87.0	99.7	100.0	100.0	1.50	84.6	97.3	99.6	100.0
83.6	99.3	100.0	100.0	1.40	80.1	94.9	98.9	99.7
78.2	97.9	100.0	100.0	1.30	75.2	90.9	96.7	99.0
75.1	96.6	100.0	100.0	1.25	71.8	87.0	94.3	97.6
70.7	93.9	100.0	100.0	1.20	67.6	82.3	90.5	95.1
66.6	89.5	99.8	100.0	1.15	65.9	75.8	85.1	90.1
60.9	81.2	98.8	100.0	1.10	58.2	67.7	76.2	80.9
53.3	66.3	92.7	100.0	1.05	54.2	57.2	60.6	63.8
1.0	2.0	3.0	4.0	skewness	1.0	2.0	3.0	4.0

Table 7.2: Estimated probabilities (%) that the p-value of the WMW test is smaller than that of the t-test.

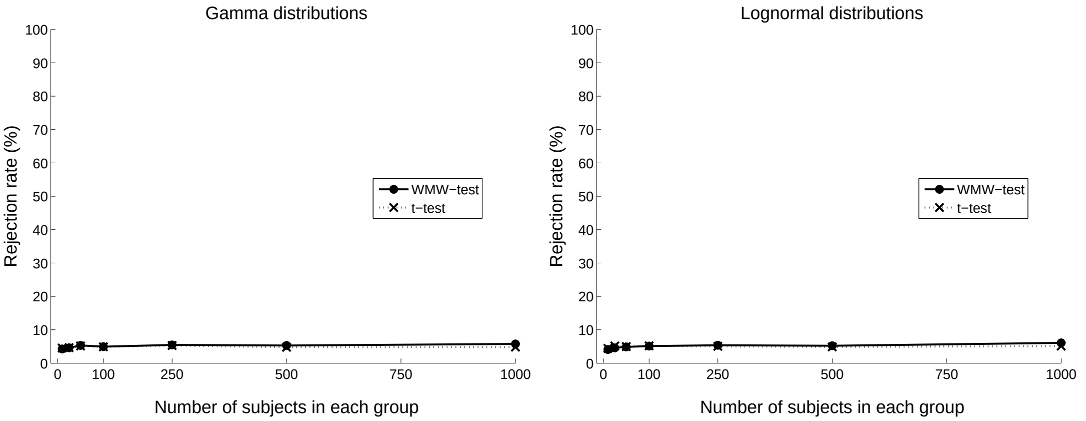

Figure 1: Rejection rates ($p < 0.05$) for a standard deviation ratio of 1.05 and a skewness of 1.0.

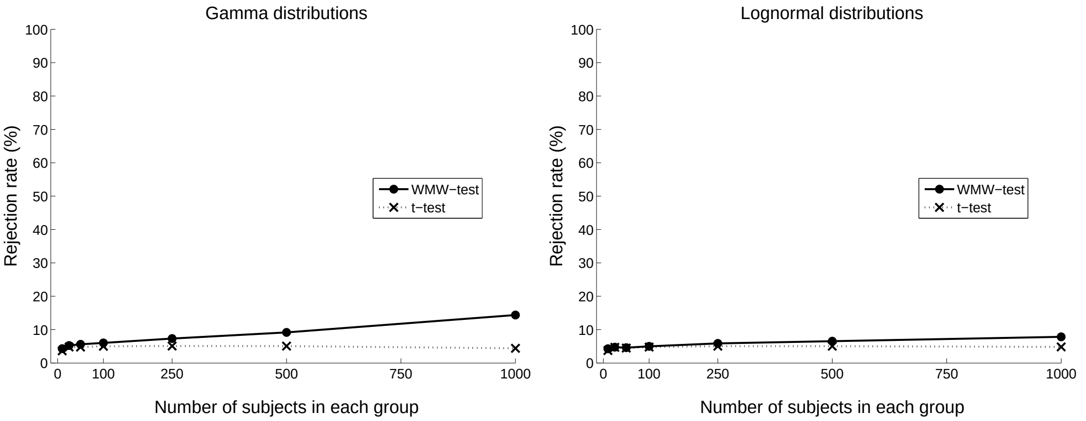

Figure 2: Rejection rates ($p < 0.05$) for a standard deviation ratio of 1.05 and a skewness of 2.0.

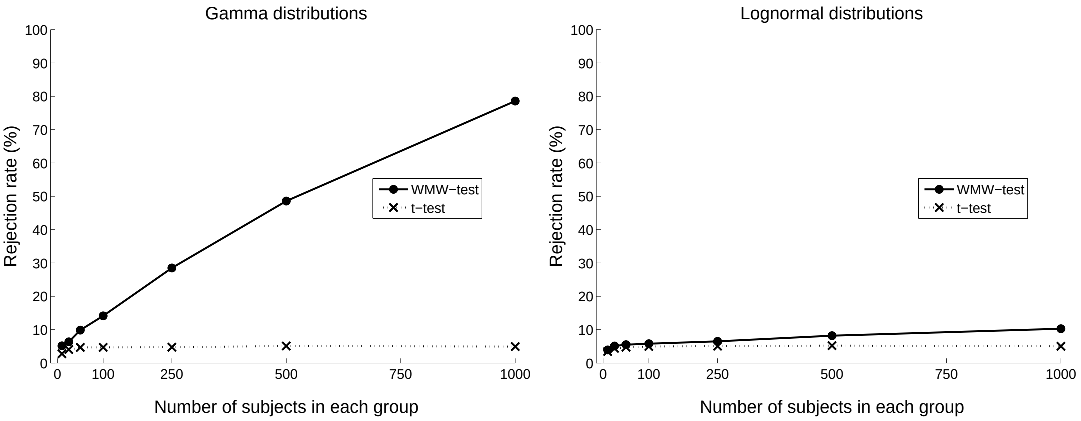

Figure 3: Rejection rates ($p < 0.05$) for a standard deviation ratio of 1.05 and a skewness of 3.0.

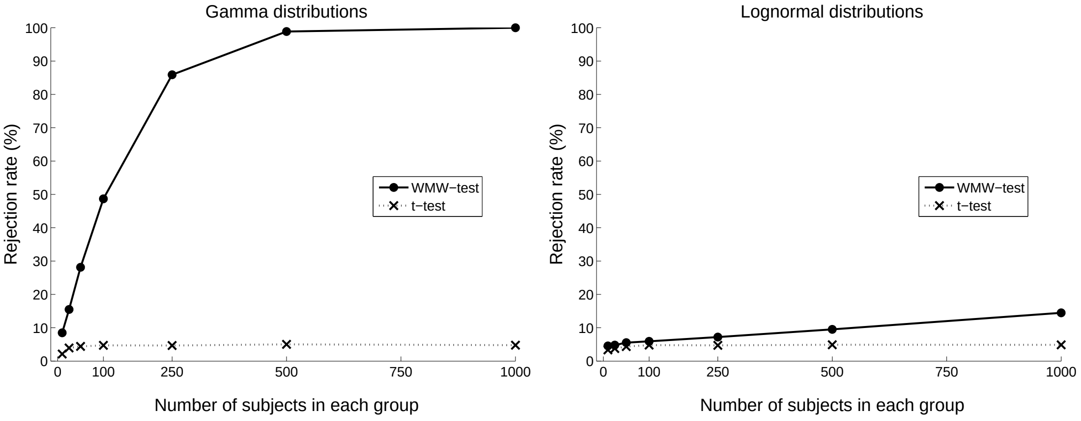

Figure 4: Rejection rates ($p < 0.05$) for a standard deviation ratio of 1.05 and a skewness of 4.0.

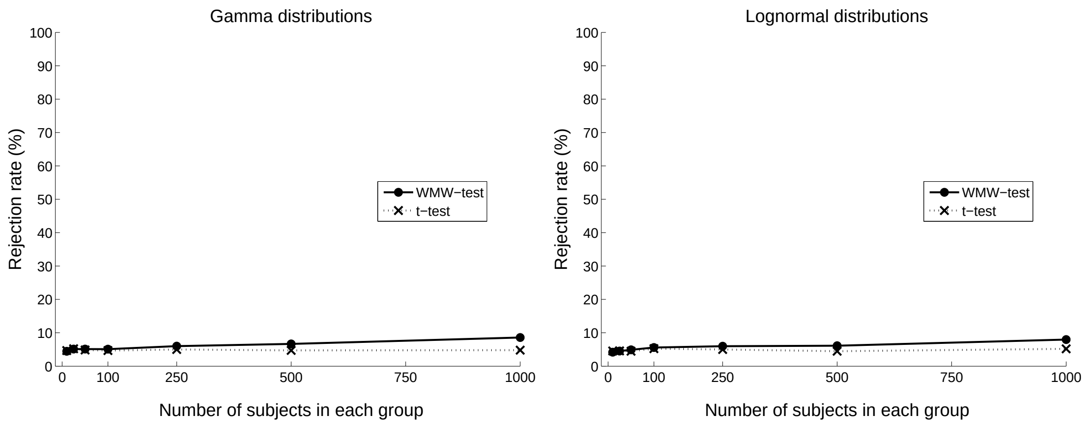

Figure 5: Rejection rates ($p < 0.05$) for a standard deviation ratio of 1.10 and a skewness of 1.0.

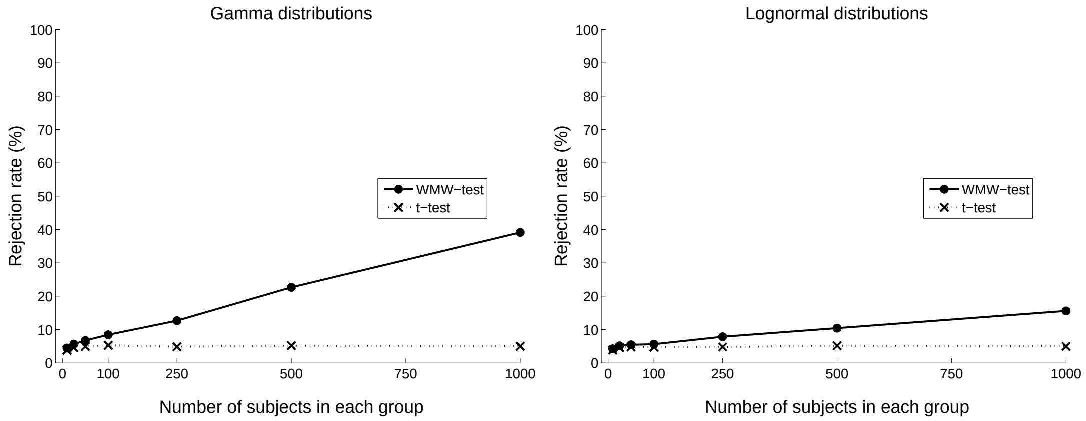

Figure 6: Rejection rates ($p < 0.05$) for a standard deviation ratio of 1.10 and a skewness of 2.0.

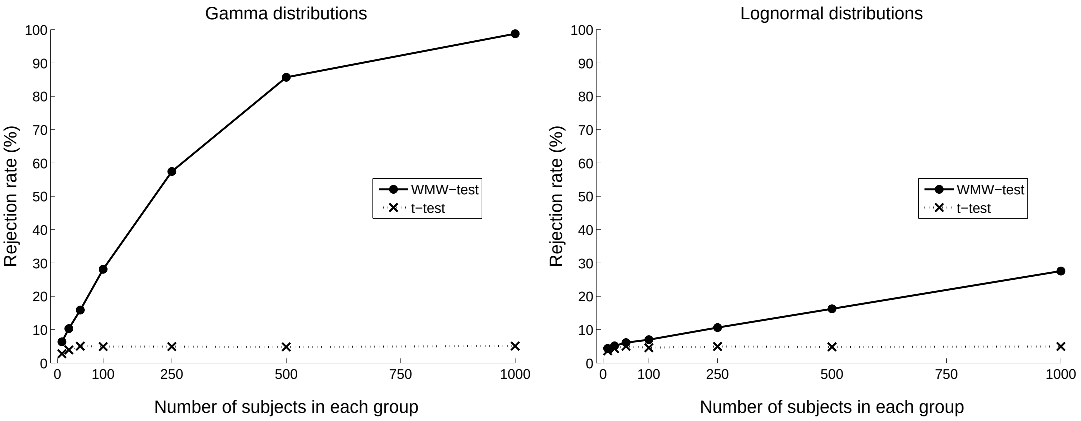

Figure 7: Rejection rates ($p < 0.05$) for a standard deviation ratio of 1.10 and a skewness of 3.0.

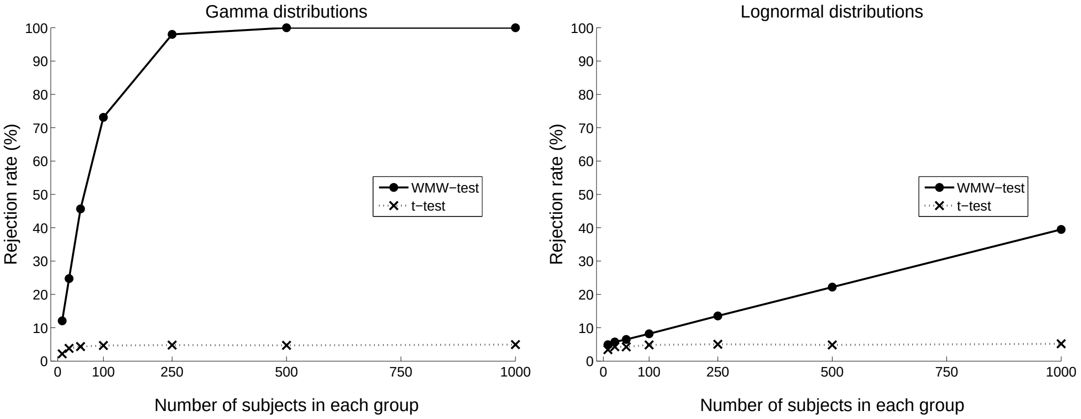

Figure 8: Rejection rates ($p < 0.05$) for a standard deviation ratio of 1.10 and a skewness of 4.0.

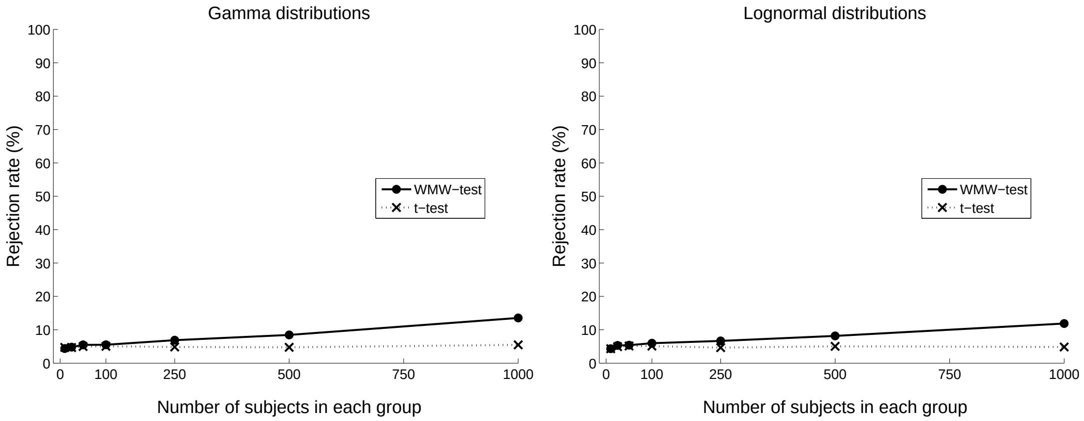

Figure 9: Rejection rates ($p < 0.05$) for a standard deviation ratio of 1.15 and a skewness of 1.0.

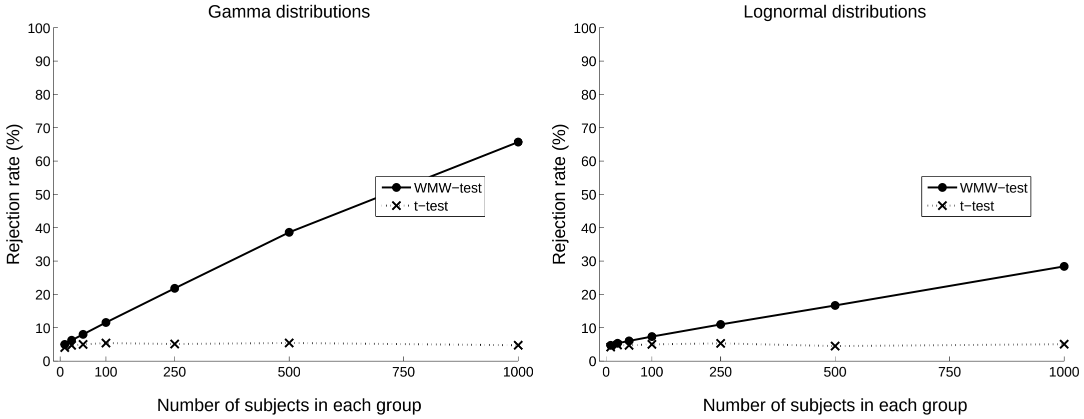

Figure 10: Rejection rates ($p < 0.05$) for a standard deviation ratio of 1.15 and a skewness of 2.0.

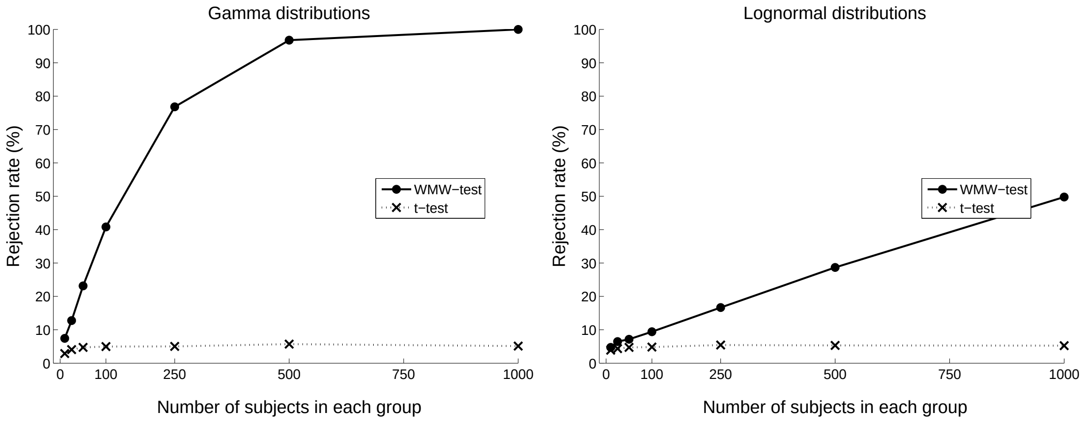

Figure 11: Rejection rates ($p < 0.05$) for a standard deviation ratio of 1.15 and a skewness of 3.0.

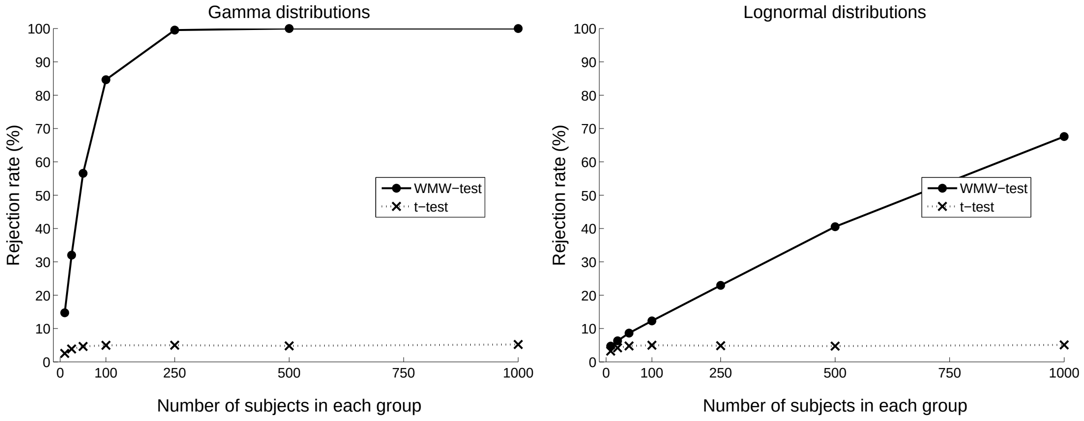

Figure 12: Rejection rates ($p < 0.05$) for a standard deviation ratio of 1.15 and a skewness of 4.0.

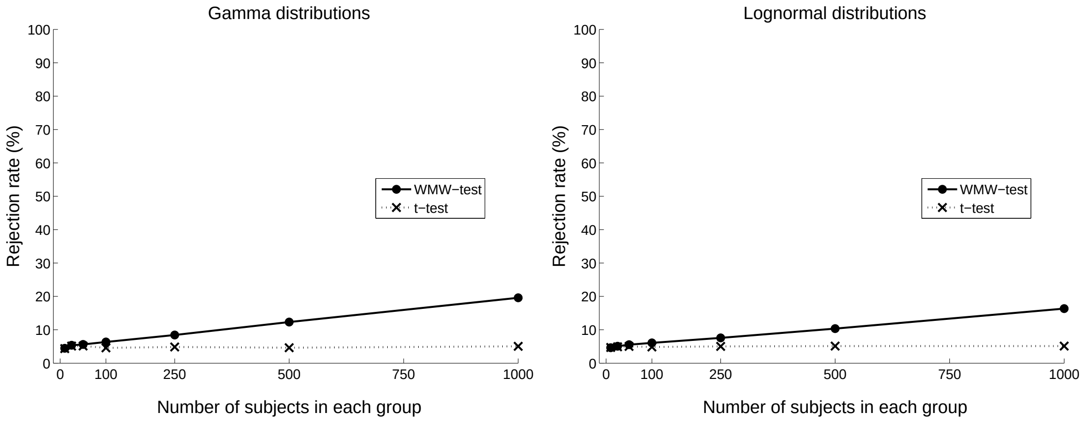

Figure 13: Rejection rates ($p < 0.05$) for a standard deviation ratio of 1.20 and a skewness of 1.0.

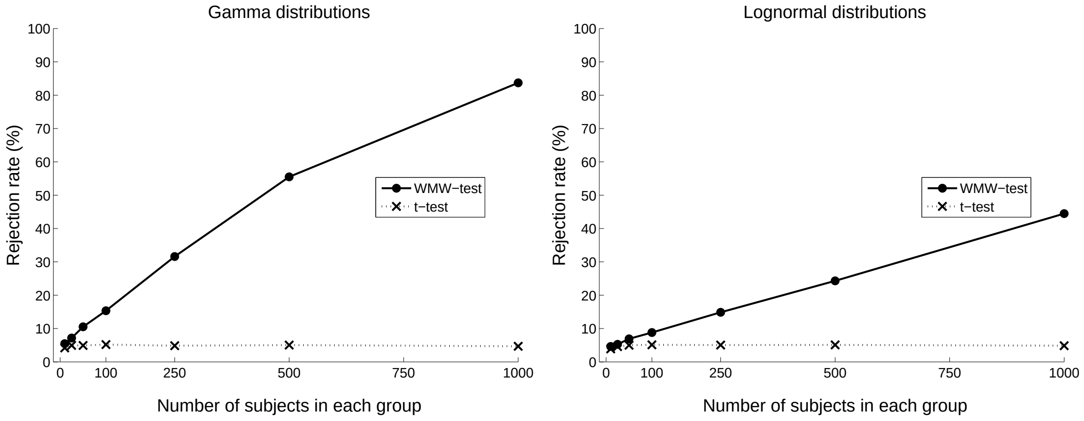

Figure 14: Rejection rates ($p < 0.05$) for a standard deviation ratio of 1.20 and a skewness of 2.0.

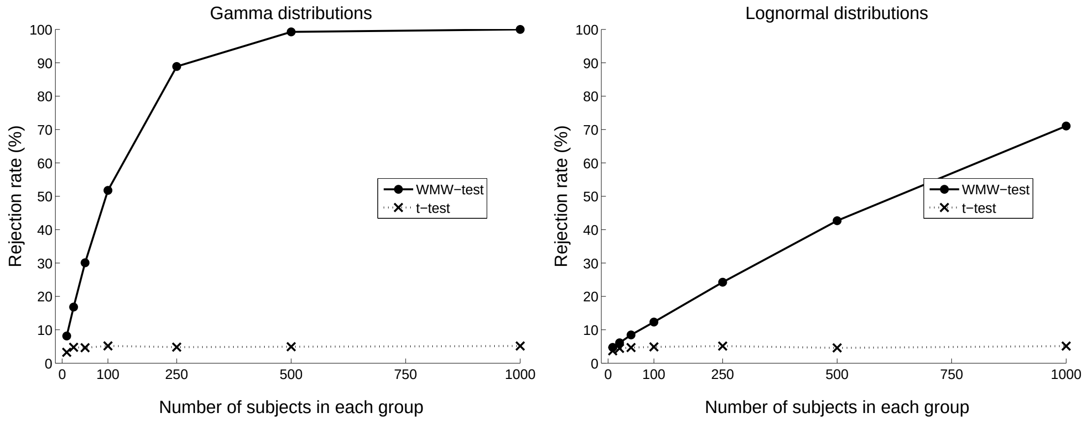

Figure 15: Rejection rates ($p < 0.05$) for a standard deviation ratio of 1.20 and a skewness of 3.0.

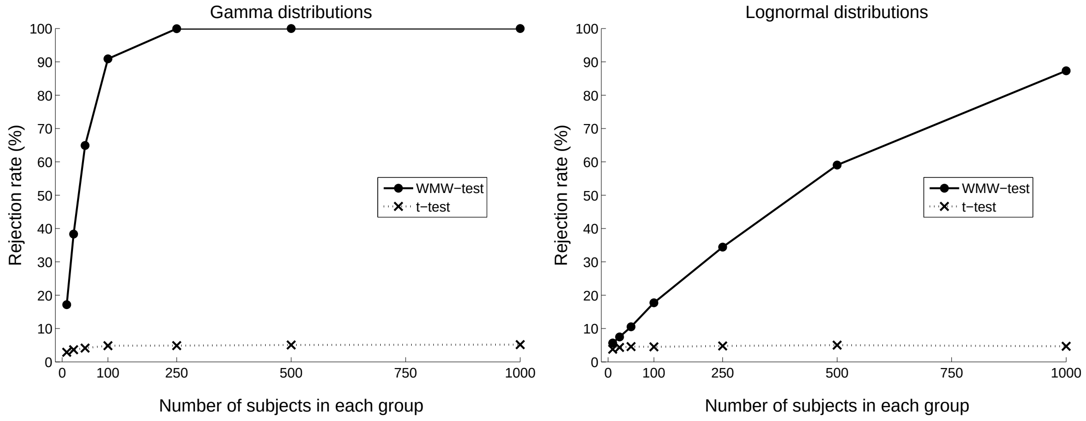

Figure 16: Rejection rates ($p < 0.05$) for a standard deviation ratio of 1.20 and a skewness of 4.0.

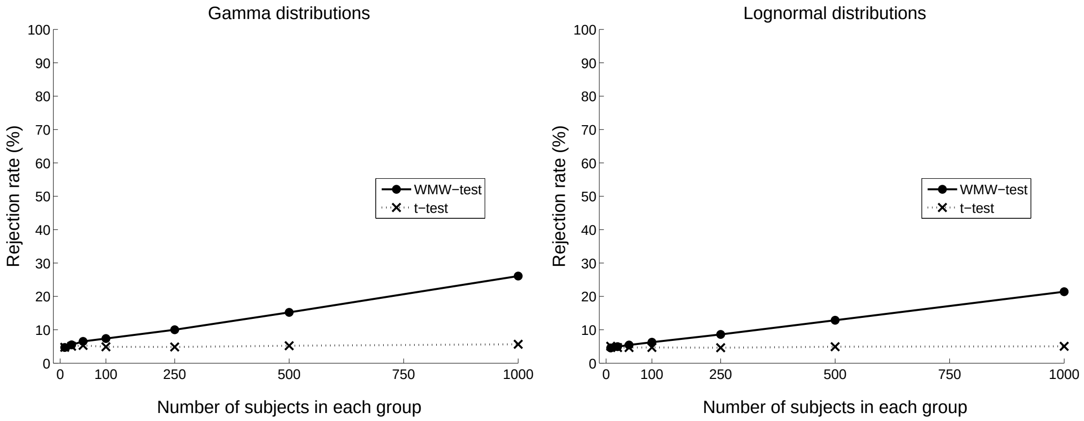

Figure 17: Rejection rates ($p < 0.05$) for a standard deviation ratio of 1.25 and a skewness of 1.0.

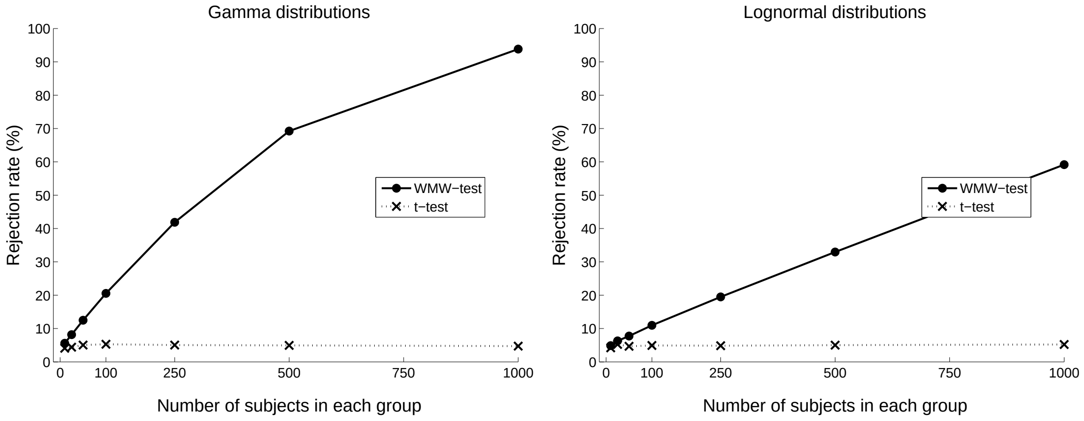

Figure 18: Rejection rates ($p < 0.05$) for a standard deviation ratio of 1.25 and a skewness of 2.0.

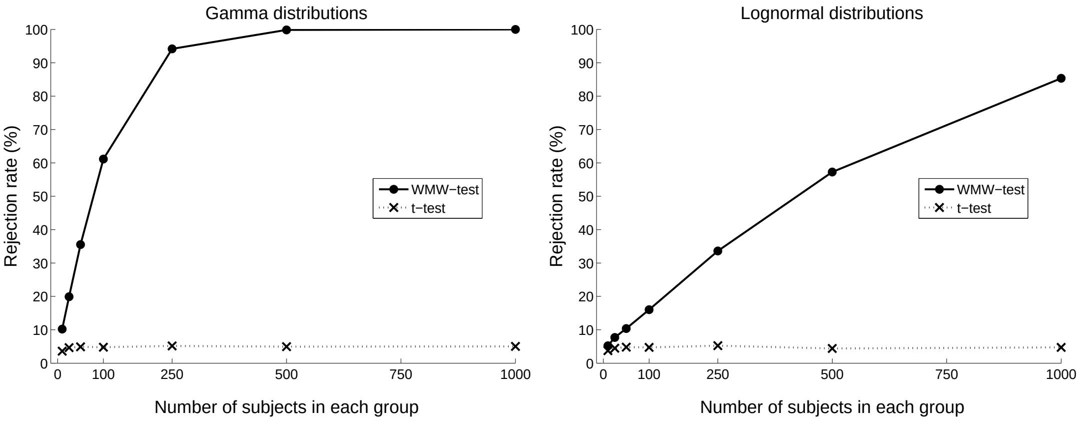

Figure 19: Rejection rates ($p < 0.05$) for a standard deviation ratio of 1.25 and a skewness of 3.0.

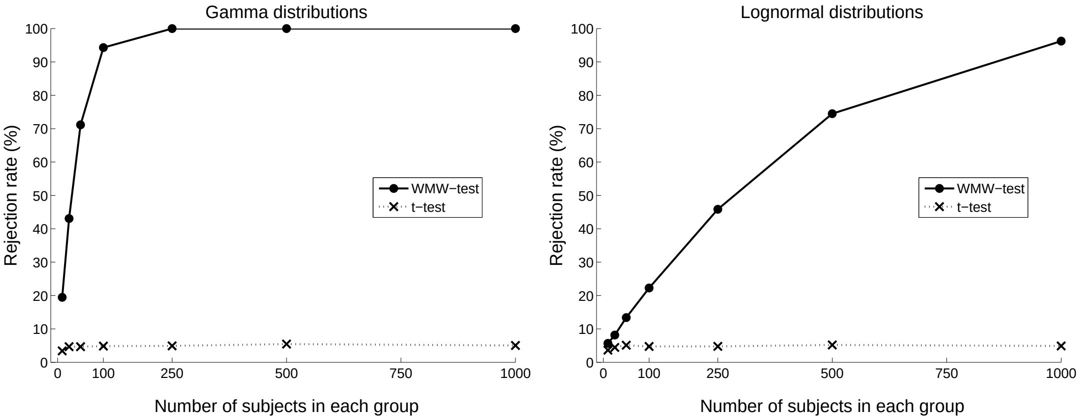

Figure 20: Rejection rates ($p < 0.05$) for a standard deviation ratio of 1.25 and a skewness of 4.0.

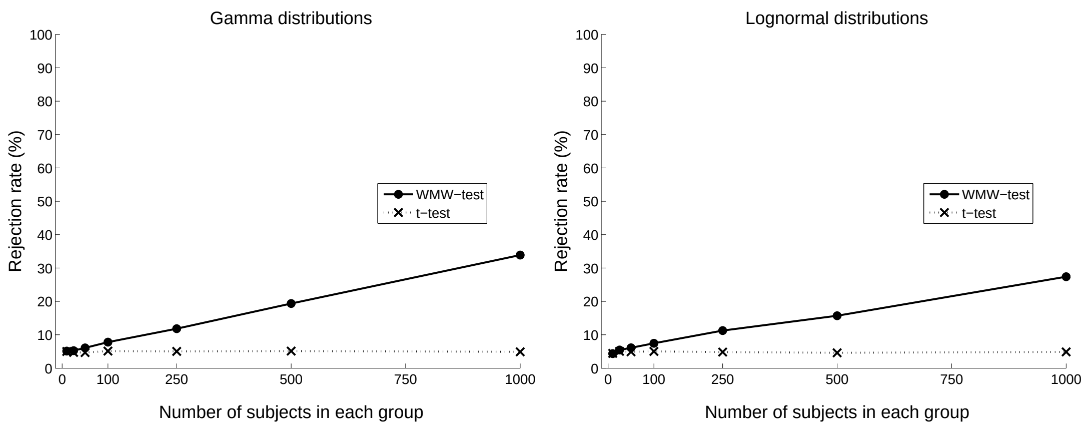

Figure 21: Rejection rates ($p < 0.05$) for a standard deviation ratio of 1.30 and a skewness of 1.0.

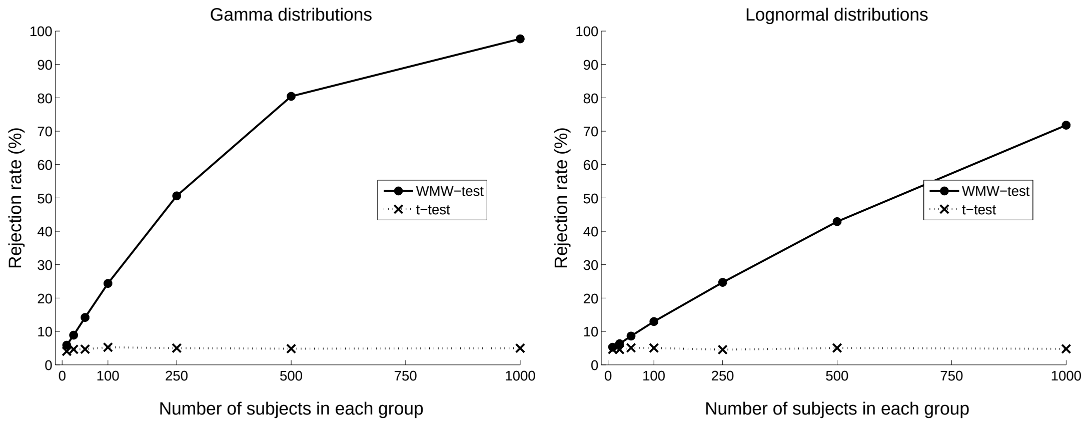

Figure 22: Rejection rates ($p < 0.05$) for a standard deviation ratio of 1.30 and a skewness of 2.0.

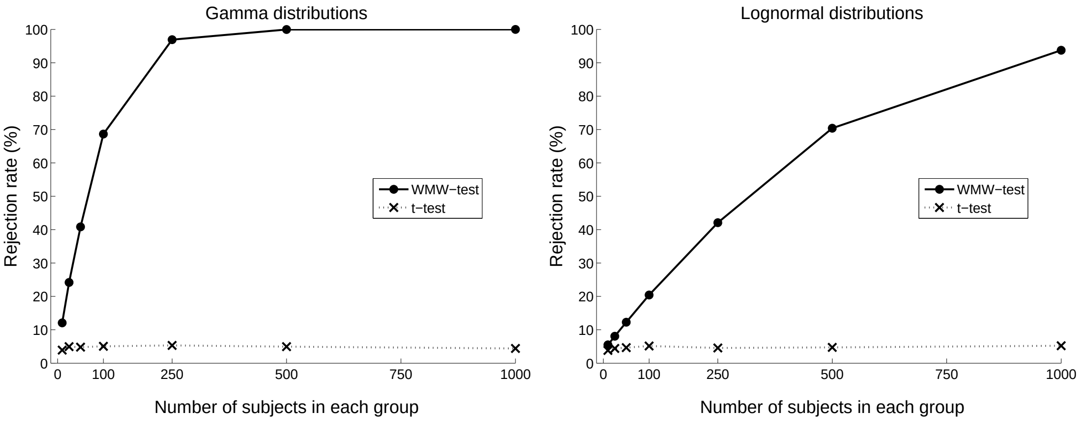

Figure 23: Rejection rates ($p < 0.05$) for a standard deviation ratio of 1.30 and a skewness of 3.0.

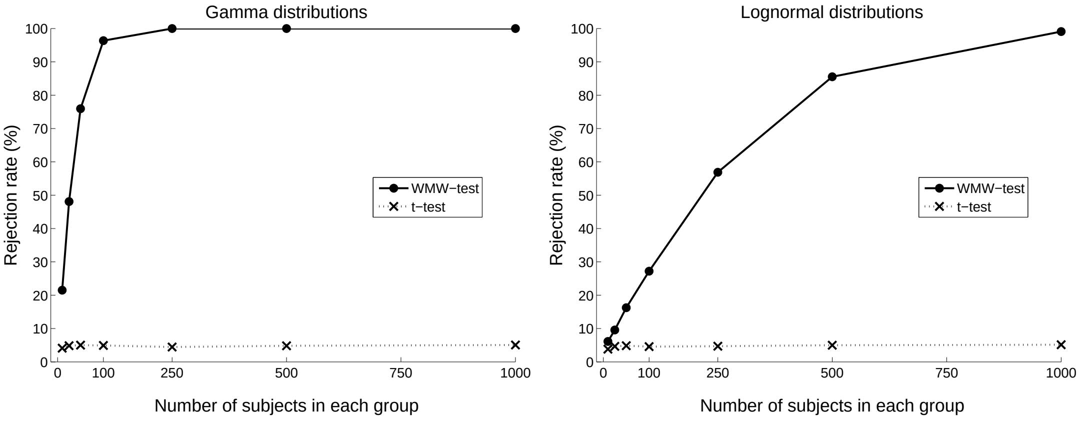

Figure 24: Rejection rates ($p < 0.05$) for a standard deviation ratio of 1.30 and a skewness of 4.0.

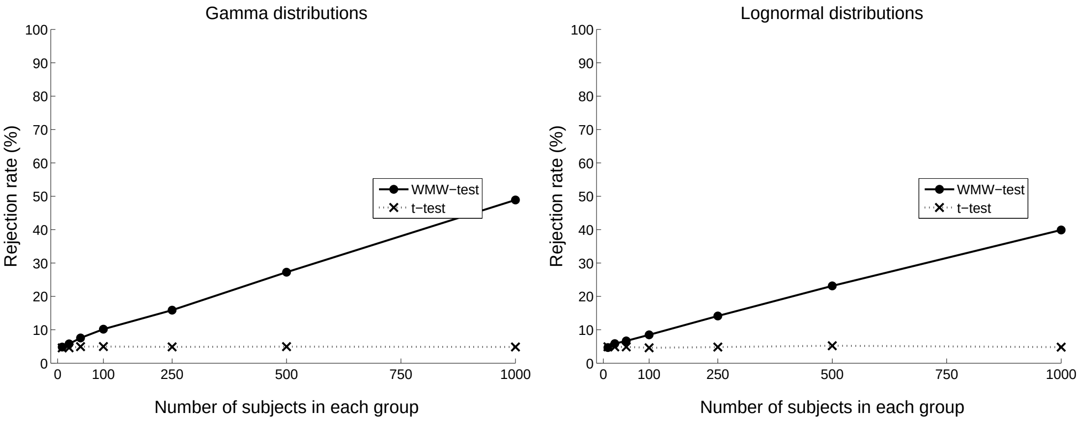

Figure 25: Rejection rates ($p < 0.05$) for a standard deviation ratio of 1.40 and a skewness of 1.0.

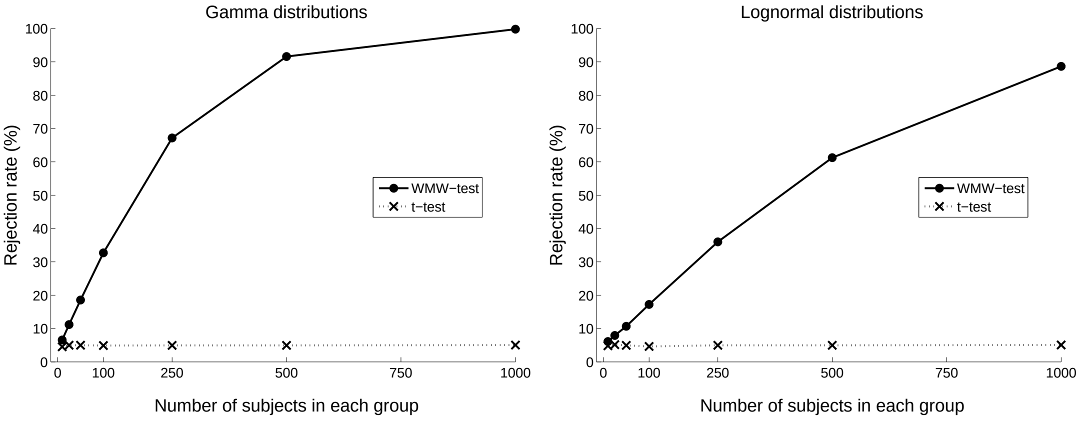

Figure 26: Rejection rates ($p < 0.05$) for a standard deviation ratio of 1.40 and a skewness of 2.0.

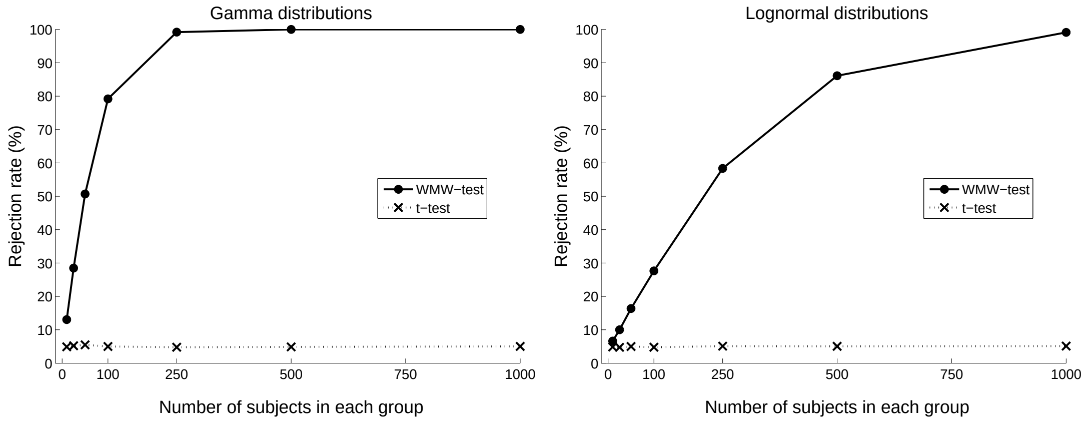

Figure 27: Rejection rates ($p < 0.05$) for a standard deviation ratio of 1.40 and a skewness of 3.0.

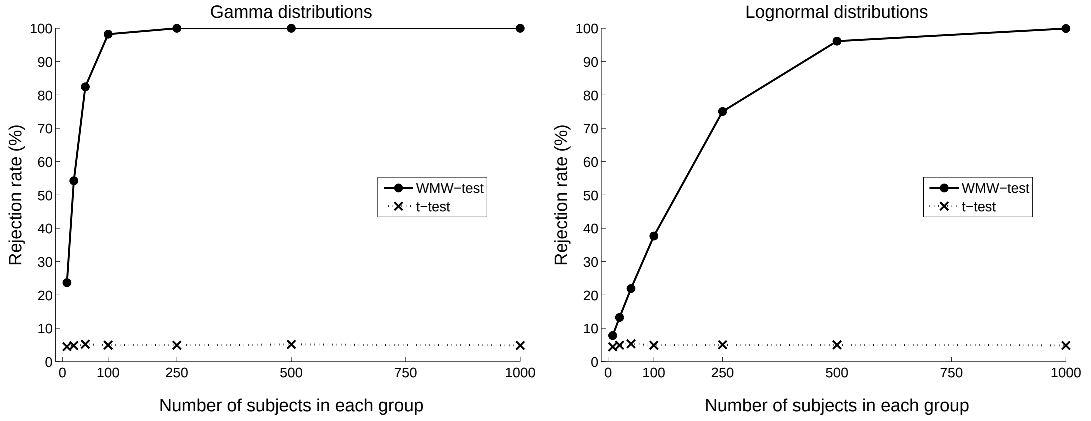

Figure 28: Rejection rates ($p < 0.05$) for a standard deviation ratio of 1.40 and a skewness of 4.0.

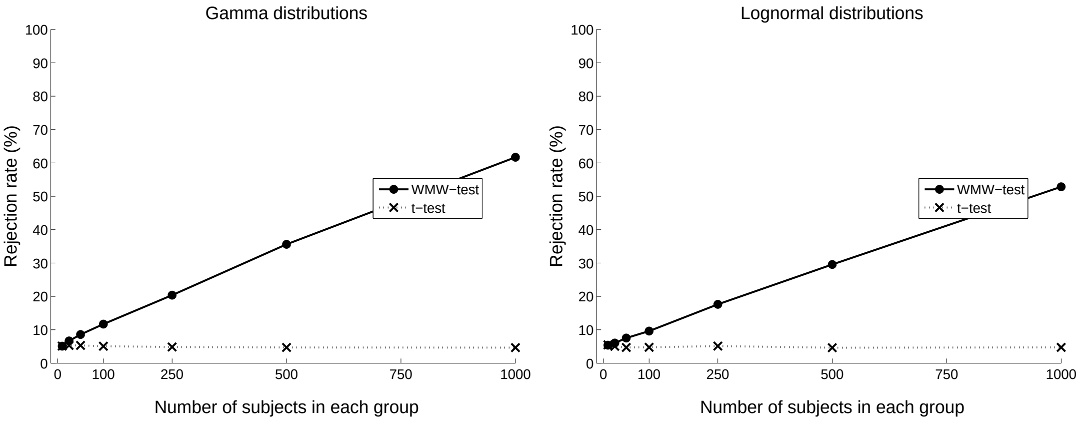

Figure 29: Rejection rates ($p < 0.05$) for a standard deviation ratio of 1.50 and a skewness of 1.0.

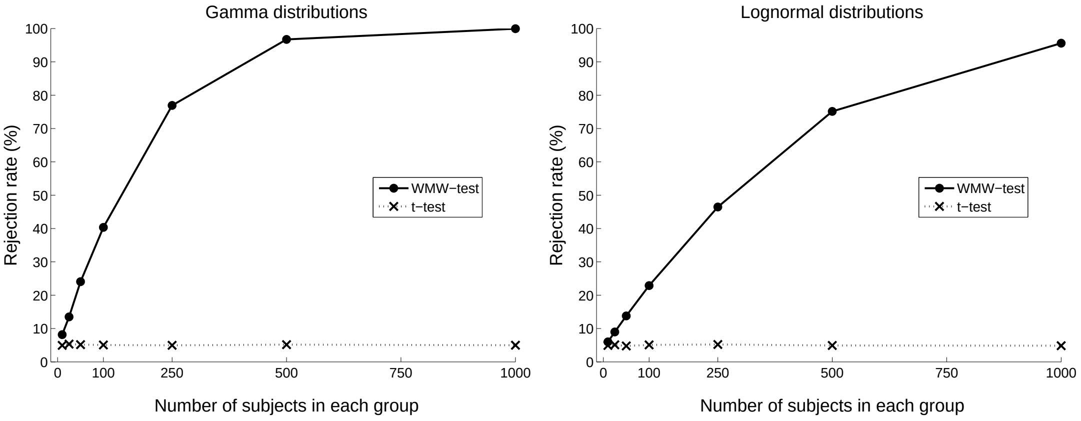

Figure 30: Rejection rates ($p < 0.05$) for a standard deviation ratio of 1.50 and a skewness of 2.0.

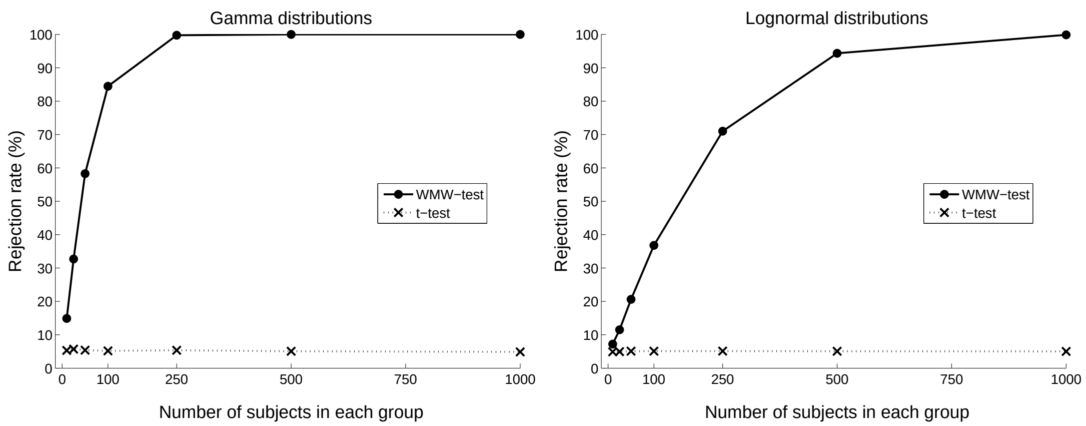

Figure 31: Rejection rates ($p < 0.05$) for a standard deviation ratio of 1.50 and a skewness of 3.0.

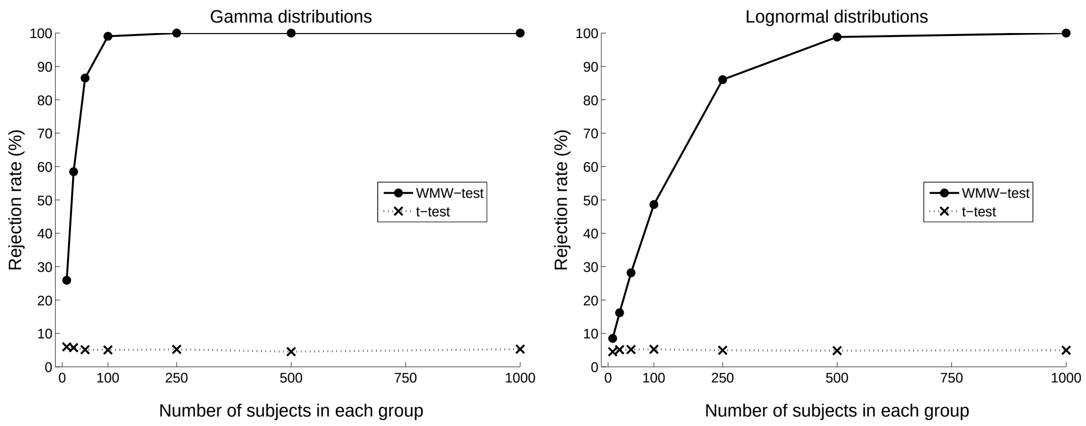

Figure 32: Rejection rates ($p < 0.05$) for a standard deviation ratio of 1.50 and a skewness of 4.0.
